# Characteristics and a comparison of the gut microbiota in two frog species at the beginning and end of hibernation

**DOI:** 10.3389/fmicb.2023.1057398

**Published:** 2023-05-03

**Authors:** Qing Tong, Wen-jing Dong, Ming-da Xu, Zong-fu Hu, Peng Guo, Xiao-yun Han, Li-yong Cui

**Affiliations:** ^1^School of Biology and Agriculture, Jiamusi University, Jiamusi, China; ^2^College of Veterinary Medicine, Northeast Agricultural University, Harbin, China; ^3^Hejiang Forestry Research Institute of Heilongjiang Province, Jiamusi, China

**Keywords:** gut microbiota, fasting, hibernation, amphibians, *Rana dybowskii*, *Rana amurensis*, season, core microbiota

## Abstract

Season has been suggested to contribute to variation in the gut microbiota of animals. The complicated relationships between amphibians and their gut microbiota and how they change throughout the year require more research. Short-term and long-term hypothermic fasting of amphibians may affect gut microbiota differently; however, these changes have not been explored. In this study, the composition and characteristics of the gut microbiota of *Rana amurensis* and *Rana dybowskii* during summer, autumn (short-term fasting) and winter (long-term fasting) were studied by high-throughput Illumina sequencing. Both frog species had higher gut microbiota alpha diversity in summer than autumn and winter, but no significant variations between autumn and spring. The summer, autumn, and spring gut microbiotas of both species differed, as did the autumn and winter microbiomes. In summer, autumn and winter, the dominant phyla in the gut microbiota of both species were Firmicutes, Proteobacteria, Bacteroidetes, and Actinobacteria. All animals have 10 OTUs (>90% of all 52 frogs). Both species had 23 OTUs (>90% of all 28 frogs) in winter, accounting for 47.49 ± 3.84% and 63.17 ± 3.69% of their relative abundance, respectively. PICRUSt2 analysis showed that the predominant functions of the gut microbiota in these two *Rana* were focused on carbohydrate metabolism, Global and overview maps, Glycan biosynthesis metabolism, membrane transport, and replication and repair, translation. The BugBase analysis estimated that among the seasons in the *R. amurensis* group, Facultatively_Anaerobic, Forms_Biofilms, Gram_Negative, Gram_Positive, Potentially_Pathogenic were significantly different. However, there was no difference for *R. dybowskii*. The research will reveal how the gut microbiota of amphibians adapts to environmental changes during hibernation, aid in the conservation of endangered amphibians, particularly those that hibernate, and advance microbiota research by elucidating the role of microbiota under various physiological states and environmental conditions.

## Introduction

The digestive systems of vertebrates are inhabited by diverse microbial communities that are critical to the health of the host (Jackson and Ultsch, 2010; Carey et al., 2013; Comizzoli et al., 2021; Perlman et al., 2022). The gut microbiota of the host mediates diverse processes, including digestion, innate immunity, vitamin synthesis, and structural and functional maturation of the gut (Bäckhed et al., 2015; Martinez-Mota et al., 2020). The gut microbiota and host have a coevolved mutualistic relationship (Sanders et al., 2014). This close relationship causes the host and microbes to change together, thereby subjecting the gastrointestinal tract to anatomical and physiological evolution (Trevelline and Kohl, 2022). However, most gut microbiota studies have been conducted on humans and mammals (Kohl and Yahn, 2016), whereas the gut microbial ecology of amphibians has received relatively less attention (Jiménez and Sommer, 2016; Chen et al., 2022).

Season has been suggested to contribute to variation in the gut microbiota of animals (Tang et al., 2019; Xiao et al., 2019; Huang and Liao, 2021; Zhou et al., 2021; Fan et al., 2022; Greene et al., 2022). Many environmental factors, such as temperature, diet, and habitat, among others, are responsible for these alterations (Xiao et al., 2019; Huang and Liao, 2021; Zhou et al., 2021; Fan et al., 2022; Greene et al., 2022). Seasonal food availability affects amphibians’ gut microbiome (Baniel et al., 2021). In temperate regions, for example, animal gut microbiota has been observed to undergo seasonal changes in response to changes in diet associated with the availability of diet (Chang et al., 2016). The type of diet and quantity of food consumed significantly affect the make-up of gut microorganisms (Xiao et al., 2019). Ectothermic amphibians have environmental body temperatures. Temperature fluctuations can alter gut bacteria growth and survival (Tong et al., 2020b). Environment can also change gut microbiome. Frogs may encounter various microbes when breeding from land to aquatic settings. Reproduction’s energy demands and hormone fluctuations may also impact amphibians’ gut microbiome (Comizzoli et al., 2021). Research into the gut microbiota of amphibians is a rapidly expanding field in which much remains to be discovered (Jiménez and Sommer, 2016; Chen et al., 2022). In particular, more research is needed to fully understand the complex interactions between amphibians and their gut bacteria, and how these interactions may vary throughout the year.

Hibernation fasting is essential for temperate frogs because it allows them to endure severe winter temperatures when food is scarce, conserves energy, and protects them from predators (Zhou et al., 2021; Regan et al., 2022). The effects of short-term hypothermic fasting of hibernation on amphibians may be different from the effects of long-term hypothermic fasting on amphibians. Fasting affects the gut microbiota and depends heavily on the host diet for metabolic substrates (Sommer et al., 2016; Tang et al., 2019). Microbes can also utilize host-derived substrates, although host food is the primary source of substrates for microbial growth (Dill-McFarland et al., 2014). Gut microorganisms have little to no access to nutritional substrates while the host is fasting. Lengthening the host’s fasting phase may cause the growth of gut microorganisms to be selectively attracted to microbial communities that can breakdown substrates derived from the host (e.g., mucin) (Xiao et al., 2019). The gut is the first organ system directly affected by dietary changes and is highly adaptable to diverse dietary situations (Cramp and Franklin, 2005). The structure and function of amphibian digestive organs undergo profound changes during the process of adapting to changes in the physiological and nutritional status during hibernation (Wiebler et al., 2018; Tong et al., 2020b). The gut microbiota in the early stages of hibernation may transition from adaptation to feeding to adaptive fasting, whereas the gut microbiota in the later stages of hibernation may be fully adapted to fasting (Tang et al., 2019). Short-term and long-term hypothermic fasting of amphibians may affect gut microbiota differently; however, these changes have not been investigated.

*Rana amurensis* and *Rana dybowskii* inhabit similar habitats (Tong et al., 2019). In summer, *R. amurensis* and *R. dybowskii* are spread across different areas to procure greater food resources (Che et al., 2007; Chen and Lu, 2011). However, these two *Rana* species are related in terms of their trophic locations, and their primary food source is insects (Xu et al., 2010). In autumn, *R. dybowskii* moved from the terrestrial to the aquatic environment, and *R. amurensis* lives near ponds where the host is in a different environmental flora and the host may select for the bacteria required in the environment (Tong et al., 2023). Both *R. amurensis* and *R. dybowskii* move to wintering ponds as soon as the temperature decreases with the arrival of autumn to begin a six-month period of hibernation (Tong et al., 2019). It is anticipated that the species and abundance of bacterial phyla, families, and OTUs will vary between seasons and during the beginning and end of hibernation; do they increase or decrease, and how many of them are consistent between the two frog species? In this study, we describe taxonomic alterations in the gut microbiotas of frogs in different seasons, and we used PICRUSt2 and BugBase analysis to infer the microbiota’s function. We propose three hypotheses: a core microbiota for both frogs during hibernation; significant differences in microbiota composition and alpha diversity between the two frog species at the beginning (autumn) and end of hibernation (spring); and significant differences in microbiota composition and alpha diversity among summer, autumn and winter seasons.

## Materials and methods

### Sample collection

Before sample collection, all animal protocols were approved by the Institutional Animal Care and Use Committee of Northeast Agricultural University (IACUC #2015-035). The accepted procedures and rules were followed for each experiment.

We sampled six separate groups of frogs; three groups were sampled from summer (15 June, natural diet in summer), autumn (15 October, 0.5 months hypothermic fasting) and spring (5 April, 6 months hypothermic fasting) *R. amurensis*, and three groups were sampled from summer, autumn and spring *R. dybowskii*. These frog samples were assigned the following labels: as (8 samples, natural diet in summer), aa (5 samples, hibernated for 0.5 months), ah (14 samples, hibernated for 6 months), ds (6 samples, normal diet in summer), da (5 samples, hibernated for 0.5 months), and dh (14 samples, hibernated for 6 months).

Frogs from groups aa (a1–a5, 15 October 2016), ah (ah1–ah7, 5 April 2017, ah8–ah14, 1 April 2018) were captured from the same natural overwintering dormant pool in Heilongjiang Province, China (47.6466 N, 130.3435 E, 98 m asl). The as group (summer gut microbiota) was sampled on 15 June 2022, in approximately 50 m of grass near the overwintering pond. Frogs from groups da (da1–da5, 15 October 2016) and dh (hibernated for 6 months, dh1–dh7, 5 April 2017, dh8–dh14, 1 April 2018) were captured from the same natural overwintering dormant pool in Heilongjiang Province, China (47.6466 N, 130.3435 E, 98 m asl). The ds group (summer gut microbiota) was sampled on 15 June 2017 in a 3.5 km forested area near the overwintering pond. In autumn the water temperature in the hibernation pond is between 3 and 4°C; in spring the water temperature in the hibernation pond is between 3 and 4°C. The frogs have been captured. The frogs were active and robust and weighed 22.32 ± 4.24 g (*R. dybowskii*) and 21.58 ± 1.93 g (*R. amurensis*).

The two frog species leave the wintering pool in April until September’s end, when they return to the wintering pool (Tong et al., 2023). In summer, *R. amurensis* inhabits grassland within about a few dozen meters of overwintering ponds. *Rana dybowskii* inhabits forested areas within a few kilometers of streams. Both species prey mostly on insects, then earthworms, mollusks, and spiders (Tong et al., 2020b). From October through April of the following year, both frog species spend around half of their lives hibernating under the ice. The water temperature for hibernation is 0-4°C. Frogs fast during hibernation and rarely move during hibernation (Tong et al., 2019). Both species fulfill a number of characteristics for the hibernation habitat they choose. Hibernation ponds where the ice does not freeze completely in winter (there are springs in the pond) and where the pond water is deep enough not to dry out, to protect them from freezing to death in winter. Most ponds have outlets and preferably long running water under the ice to ensure sufficient dissolved oxygen. Leaves, thin mud, pebbles and boulders at the bottom of the water can provide hiding places for frogs and deter natural predators from attacking them (Jackson and Ultsch, 2010).

The laboratory at Northeast Agricultural University received all samples from frogs for quick analysis of the intestinal bacterial population. Within 20 min of the frogs’ deaths, samples of their intestinal contents were taken. The following euthanasia procedure was performed: gauze was tiled in a glass dryer, and then a cotton ball soaked with a mixture of ether and alcohol was placed underneath to anaesthetise the frog; the frog’s neck was flexed, the foramen magnum was identified, and a rigid metal rod was inserted cranially and pivoted/rotated within the cranium to destroy the proximal spinal cord and brain. Prior to disposing of the euthanized frogs, death was verified by performing a physical euthanasia method or by determining that the heart had stopped beating. Then, the digestive tract was carefully isolated from the body, and the lower GI (gastrointestinal) tract contents were collected. To avoid cross-contamination, each sample was collected using a fresh pair of sterile tweezers. The contents of each intestine were emptied into a sterile vial and immediately stored at −80°C.

### DNA extraction and PCR amplification

Using the E.Z.N.A.^®^ Soil DNA Kit, DNA was extracted (Omega Bio-Tek, Norcross, GA, USA). A NanoDrop 2000 (Thermo Scientific, Wilmington, DE, USA) was used to determine the quantity and purity of the DNA, and 1% agarose gel electrophoresis was used to assess the DNA quality. Using thermocycler PCR equipment (GeneAmp 9700, ABI, USA) and the primers 338F (5’-ACTCCTACGGGAGGCAGCAG-3’) and 806R (5’-GGACTACHVGGGTWTCTAAT-3’), the V3-V4 hypervariable portions of the bacterial 16S rRNA gene were amplified. Each PCR was carried out three times. FastPfu buffer (5, 4 μl), dNTPs (2.5 mM, 2 μl), FastPfu polymerase (0.4 μl), template DNA (10 ng), and each primer (5 M, 0.8 μl) were combined in a 20:l mixture for each reaction. According to the manufacturer’s instructions, the PCR products were extracted from a 2% agarose gel, further purified using the AxyPrep DNA Gel Extraction Kit from Axygen Biosciences in the USA, and quantified using QuantiFluorTM-ST from Promega in the USA. Denaturation at 95°C for 3 min; 27 cycles of 95°C for 30 s; annealing at 55°C for 30 s; and elongation at 72°C for 45 s; and finally, a final extension at 72°C for 10 min.

### Illumina MiSeq sequencing

Pooled amplicons were sequenced in equimolar ratios on an Illumina MiSeq platform (San Diego, CA, USA) using conventional methods.

### Processing of sequencing data

Raw fastq files were demultiplexed, quality filtered with Trimmomatic and merged with FLASH according to the following criteria. First, 300-bp reads at any site with an average quality score <20 was truncated across a 50-bp sliding window such that only reads ≥50 bp were retained for analysis. Second, incorrect barcode sequences, sequences with two nucleotide mismatches in the primer, and reads with ambiguous characters were omitted. Third, only sequences with >10 bp of overlap were assembled according to the overlapping sequence, and unassembled reads were omitted. OTUs were grouped using uPARs 7.1 with a 97% similarity criterion, and chimeric sequences were removed using UCHIME. All 16S rRNA gene sequences were taxonomized against the Silva (SSU128) 16S rRNA database with a 70% confidence criterion.

### Ecological and statistical analysis

Mothur was used to analyze alpha diversity (abundance-based coverage estimators) Ace, Chao, Shannon, Simpson, and observed richness (Sobs) (Hadizadeh et al., 2017). Alpha diversity analysis was performed by FDR-corrected Kruskal–Wallis and Tukey–Kramer *post-hoc* tests. Only *P* values 0.05 are shown. A Mantel test was performed to analyze the correlation between the frog gut microbiota and the different seasons in both species. The Bray-Curtis distances and unweighted UniFrac dissimilarity index were used to create ordination graphs in R vegan (Knights et al., 2011). These matrices were analyzed using principle coordinate analysis (PCoA) and permutational multivariate analysis of variance (PERMANOVA)/Adonis in the R vegan package (Mcardle and Anderson, 2001). The core gut microbiota of the frogs was assigned if it was found in 90% of the groups and represented > 0.1% of the reads. The unique and shared OTUs are illustrated on a Venn diagram plotted using the R package. Differential OTUs were found using linear discriminant analysis (LDA) and effect size (LEfSe), which considers statistical significance and biological relevance (Zhong et al., 2015). One-way analysis of variance (ANOVA) was used to analyze population differences with a significance level of *P* < 0.05. Using the “ggtern” and “ggplot2” packages, a ternary plot was utilized to demonstrate the relative relationship and distribution of the dominating species (>0.5% in at least one sample) between the three seasons (Xie et al., 2022). We utilized SourceTracker to determine the fraction of the frog’s gut microbial community in one season that originated from other seasons. Microbial SourceTracker is a Bayesian method that is more precise than existing approaches for predicting the fraction of source habitats within a sink environment (Knights et al., 2011).

Functional shifts in the microbiota of the two frog species were predicted using PICRUSt2, which can both predict the KEGG ortholog (KO) functional profiles of microbial communities using 16S rRNA gene sequences (Langille et al., 2013) and link OTUs with gene content using a phylogenetic tree of 16S rRNA gene sequences. Thus, the PICRUSt2 forecast relies on the tree topology and the distance to the next organism; a nearest neighbor always exists, even in the case of large distances. The Kruskal–Wallis H test was used to compare changes in relative abundance among the summer, autumn and spring groups. Only differences with *P* < 0.05 are presented. BugBase^1^ is a microbiome analysis tool that identifies high level phenotypes present in microbiome samples and is capable of phenotype prediction. BugBase first normalizes the OTU by the predicted 16S copy number and then uses the provided pre-computed file to predict microbial phenotypes (Lucas et al., 2018). The phenotype types include Anaerobic, Contains_Mobile_Elements, Facultatively_Anaerobic, Forms_Biofilms, Gram_Negative, Gram_Positive, Potentially_Pathogenic, and Stress_Tolerant. The Kruskal–Wallis H test was used to compare changes in relative abundance among the summer, autumn and spring groups. Only differences with *P* < 0.05 are presented.

Data Accession numbers: The obtained raw sequences were deposited in the NCBI database (Accession numbers: PRJNA422735, PRJNA423108, PRJNA428920, PRJNA626375, and PRJNA940907).

## Results

### Alpha diversity

**FIGURE 1:**
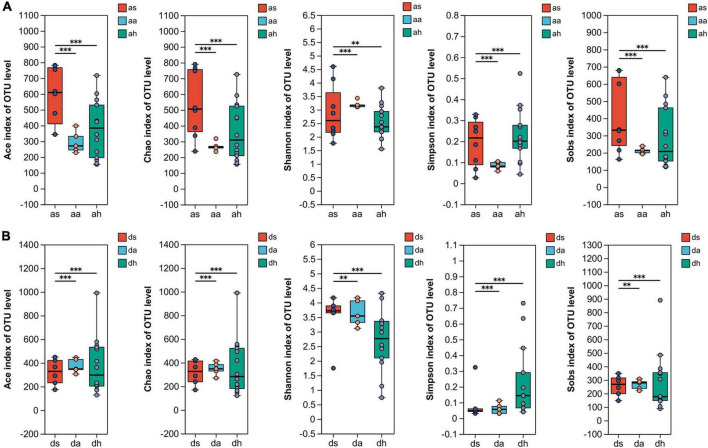
The alpha diversity of *R. amurensis* and *R. dybowskii* gut microbiotas. The alpha diversity of *R. amurensis* **(A)** and *R. dybowskii* **(B)** as measured by the Ace, Chao, Shannon, Simpson, and observed richness (Sobs) indices. Alpha diversity analysis was performed by FDR-corrected Kruskal–Wallis and Tukey–Kramer *post-hoc* tests. * stands for 0.01 < *P* ≤ 0.05, and ^**^ stands for 0.001 < *P* ≤ 0.01, ^***^ stands for *P* < 0.001.

Illumina MiSeq sequencing resulted in 2,185,735 high-quality reads. The average length of the detected sequences was 448 bp. A total of 2,371 OTUs were obtained with 97% similarity to the standard. The sequencing depth was revealed by rarefaction and Shannon curves (Supplementary Figure 1). The plateau status of the rarefaction curves indicated a sufficient sequencing depth (Supplementary Figure 1). The alpha diversity in the gut microbiota were significantly higher in summer than in autumn and winter. The Ace, Chao, Shannon, Simpson, and Sobs indices indicated significant differences in diversity among summer, autumn and spring (Kruskal–Wallis test, *P* < 0.05, all cases; Figure 1). The Ace, Chao, Shannon, Simpson, and Sobs indices showed significant diversity differences between summer and autumn and summer and spring in both frog species (Kruskal-Wallis test, *P* < 0.05, Figure 1). Both frog species indicated no significant differences in diversity between autumn and spring (Kruskal–Wallis test, *P* > 0.05, Figure 1).

### Beta diversity

**FIGURE 2:**
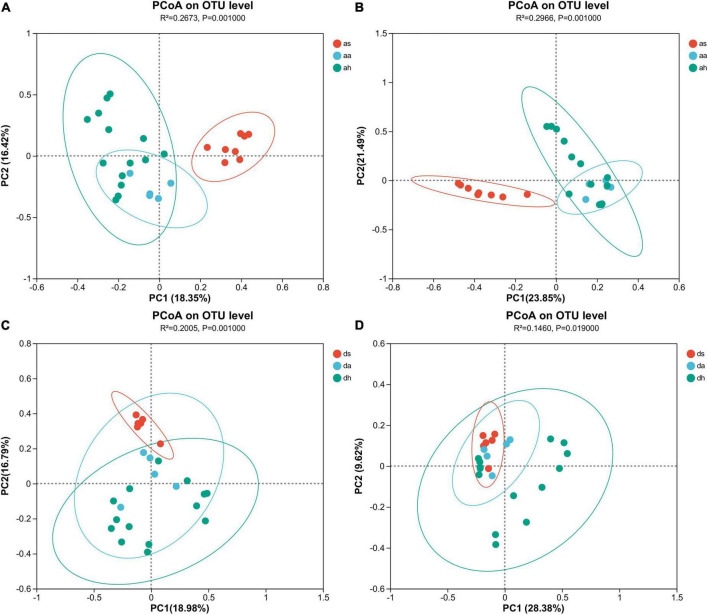
Gut microbiota differences and similarities. Principle coordinate analysis (PCoA) indicates separation by seasons based on Bray-Curtis **(A,C)** and unweighted UniFrac **(B,D)** distances. All OTUs were subjected to PCoA. Each dot represents the gut microbial community of one brown frog.

Season was a factor affecting gut microbiome. Cluster analysis showed that in *R. amurensis*, summer samples were distant from autumn and winter samples, which overlapped and clustered together (Figures 2A, B and Supplementary Figure 2). The gut microbiome of *R. amurensis* differed significantly among summer, autumn, and winter (Adonis, Bray-Curtis, *R*^2^ = 0.267, *P* = 0.001, unweighted UniFrac, *R*^2^ = 0.297, *P* = 0.001; Figures 2A, B). There were also significant variations in microbiome between summer and autumn (Adonis, Bray-Curtis, *R*^2^ = 0.273, *P* = 0.002, unweighted UniFrac, *R*^2^ = 0.419, *P* = 0.002), summer and winter (Adonis, Bray-Curtis, *R*^2^ = 0.203, *P* = 0.001, unweighted UniFrac, *R*^2^ = 0.241, and *P* = 0.001), and autumn and winter (Adonis, Bray-Curtis, *R*^2^ = 0.174, *P* = 0.002, unweighted UniFrac, *R*^2^ = 0.122, *P* = 0.020) (Figures 2A, B). The Mantel test indicated seasons was a significant predictor of microbiota composition (Mantel test; *r* = 0.347, *P* = 0.001). The Mantel test also showed significant differences between summer and autumn (Mantel test; *r* = 0.478, *P* = 0.005), summer and winter (Mantel test; *r* = 0.595, *P* = 0.001), and autumn and winter (Mantel test; *r* = 0.241, *P* = 0.011).

Season was the factor affecting the composition of gut microbiota in *R. dybowskii*. According to the results of the cluster analysis, the samples collected during the summer season of *R. dybowskii* were distinct from those collected during the autumn and winter seasons, which tended to overlap and cluster together (Figures 2C, D and Supplementary Figure 2). The gut microbiota of *R. dybowskii* changed significantly among summer, autumn, and winter (Adonis, Bray-Curtis, *R*^2^ = 0.201, *P* = 0.001, unweighted UniFrac, *R*^2^ = 0.146, *P* = 0.019; Figures 2C, D). There were also significant variations in microbiome between summer and autumn (Adonis, Bray-Curtis, *R*^2^ = 0.218, *P* = 0.003, unweighted UniFrac, *R*^2^ = 0.183, *P* = 0.003), summer and winter (Adonis, Bray-Curtis, *R*^2^ = 0.171, *P* = 0.001, unweighted UniFrac, *R*^2^ = 0.109, *P* = 0.027), and autumn and winter (Adonis, Bray-Curtis, *R*^2^ = 0.101, *P* = 0.036) (Figures 2C, D). However, there was no significant variation in the microbiome between autumn and winter (Adonis, unweighted UniFrac, R^2^ = 0.083, P = 0.110) (Figures 2C, D). The Mantel test indicated season was a significant predictor of microbiota composition (Mantel test; *r* = 0.342, *P* = 0.001). The Mantel test showed a statistically significant between summer and autumn (Mantel test; *r* = 0.441, *P* = 0.001), summer and winter (Mantel test; *r* = 0.372, *P* = 0.002). The Mantel test showed no significant differences between autumn and winter (Mantel test; *r* = 0.109, *P* = 0.333).

### The composition and SourceTracker analysis to the gut microbiota

**FIGURE 3:**
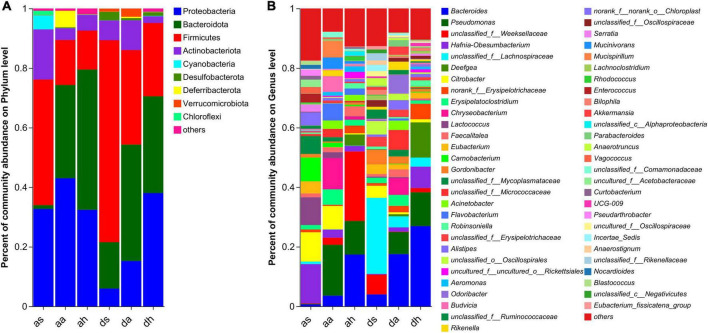
Taxonomic composition of microorganisms of *R. amurensis* and *R. dybowskii.* Bar graph study of bacterial communities at the phylum **(A)** and genera **(B)** levels. Only phyla and genera with relative abundances greater than 1% in at least one sample are displayed in this figure.

The dominant phyla (> 1%) in the gut microbiota of *R. amurensis* were Firmicutes, Proteobacteria, Bacteroidetes, Actinobacteria, Cyanobacteria, Deferribacteres, and Chloroflexi in the summer, Bacteroidetes, Proteobacteria, Firmicutes, Actinobacteria, Deferribacteres, and Verrucomicrobiota in the autumn, and Bacteroidetes, Proteobacteria, Firmicutes, Actinobacteria, and Deferribacteres in the spring (Figure 3A).

The color differences in Figure 3B indicate large differences in the percentages of the gut microbiotas. In summer, the top 10 genera in terms of abundance of the two frog species were unclassified_f__Lachnospiraceae, *Hafnia-Obesumbacterium*, *Citrobacter*, *Lactococcus*, *Carnobacterium*, *Eubacterium*, unclassified_f__Mycoplasmataceae, unclassified_f__Weeksellaceae, *Gordonibacter*, and norank_f__norank_o__Chloroplast (Figure 3B and Supplementary Figure 3). In autumn, the top 10 genera in terms of abundance of the two frog species were *Pseudomonas*, *Bacteroides*, *Chryseobacterium*, unclassified_f__Micrococcaceae, *Erysipelatoclostridium*, *Citrobacter*, *Flavobacterium*, *Odoribacter*, *Mucispirillum*, and *Acinetobacter* (Figure 3B and Supplementary Figure 3). In spring, the top 10 genera in terms of abundance of the two frog species were *Bacteroides*, *unclassified_f__Weeksellaceae*, *Pseudomonas*, *Deefgea*, *Hafnia-Obesumbacterium*, norank_f__Erysipelotrichaceae, *Faecalitalea*, *Robinsoniella*, uncultured_f__uncultured_o__Rickettsiales, and unclassified_f__Lachnospiraceae (Figure 3B and Supplementary Figure 3).

**FIGURE 4:**
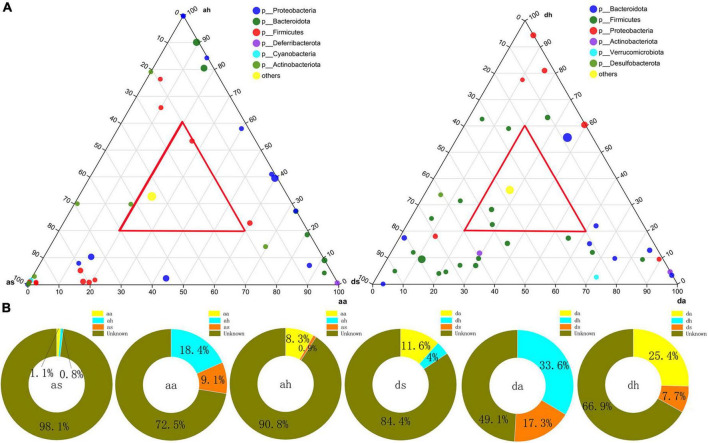
Distributions of dominant OTUs and SourceTracker analysis to the gut bacterial community. **(A)** Ternary plots showing the distributions of dominant OTUs among summer, autumn, and spring gut at the *R. amurensis* and *R. dybowskii*. The sizes of the circle are proportional to the relative abundance of OTUs. **(B)** SourceTracker analysis of the relative contribution of microbiota sources to the gut bacterial community of *R. amurensis* and *R. dybowskii*.

At the finer taxon level, the ternary plots demonstrated the distributions of dominant species (relative abundance >0.5%) across the three habitats at the *R. amurensis* or *R. dybowskii*, respectively (Figure 4A). Only one and two dominant taxa were shared among the three seasons (red triangle) at the *R. amurensis* or *R. dybowskii*, respectively (Figure 4A). In contrast, more diverse taxa were unique in each season in both species (Figure 4A). Thus, only a few dominant taxa were ubiquitous across the three seasons (Figure 4A). We used SourceTracker analysis to find the source of the gut microbiota and found that the summer microbiota of both species was of unknown, and very little in autumn and the adjacent season winter; the autumn microbiota of both species was mainly of unknown, with a proportion in summer (as 9.1%, ds 17.3%) and winter (ah 18.4, dh 33.6%); and the winter microbiota of both species was mainly of unknown, with a proportion in summer (aa: as 8.3%, da 25.4%) and autumn (as 0.9%, ds 7.7%) (Figure 4B).

### The variation of frog gut microbiotas

**FIGURE 5:**
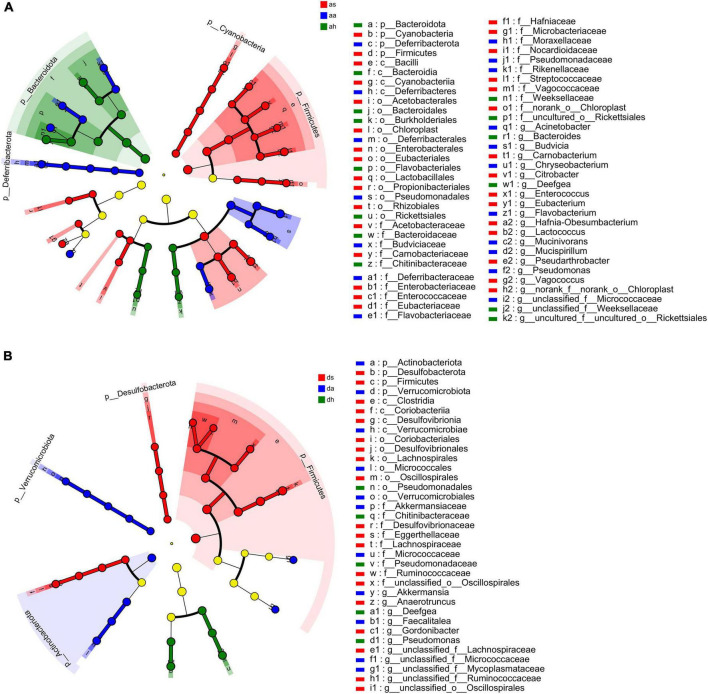
LEfSe analysis of *R. amurensis* and *R. dybowskii* gut bacterial biomarkers among three seasons. Treatment color distinguished as/ds, aa/da, and ah/dh groups [as/ds for summer samples, aa/da for autumn samples, and ah/dh for spring samples of *R. amurensis* **(A)**/*R. dybowskii* **(B)**]. Each circle’s diameter reflects its richness. Multiclass analysis is flexible (at least one class differential). Inside-out circles reflect domain to genus taxonomy. Inside-out circles symbolize phylum to genus taxonomy. Class, order, and family labels appear. All taxa with an LDA score > 4 are shown.

Thirty-four phyla were identified in *R. amurensis* in different seasons, and 10 phyla (Bacteroidota, Campylobacterota, Chloroflexi, Cyanobacteria, Deferribacterota, Deinococcota, Desulfobacterota, Firmicutes, Fusobacteriota, and Patescibacteria) showed significant differences (Kruskal–Wallis test, *P* < 0.05). The differences in the relative abundances of bacterial taxa (from the phylum to genus level) across frogs were estimated using LEfSe with the aim of identifying changes in the microbial community compositions among different seasons (Figure 5A). At the phylum level, significant enrichment of four phyla (Cyanobacteria, Chloroflexi, Firmicutes, and Patescibacteria) in summer, and of three phyla (Deferribacterota, Desulfobacterota, and Fusobacteriota) in autumn, and of three phyla (Actinobacteria, Firmicutes, and Tenericutes) in spring was found in in in *R. dybowskii* (LDA > 4, *P* < 0.05; Figure 5A). Of all 742 genera in *R. amurensis*, 269 showed significant differences among different seasons (Kruskal-Wallis test, *P* < 0.05). At the genus level, significant enrichment of nine genera (*Carnobacterium*, *Citrobacter*, *Enterococcus*, *Eubacterium*, *Hafnia-Obesumbacterium*, *Lactococcus*, *norank_f__norank_o__Chloroplast, Pseudarthrobacter*, and *Vagococcus*) in summer, and of nine genera (*Acinetobacter, Budvicia*, *Chryseobacterium*, *Flavobacterium*, *Mucinivorans*, *Mucispirillum*, *Pseudomonas*, *Rikenella*, and unclassified_f__Micrococcaceae) in autumn, and four genus (*Bacteroides*, *Deefgea*, unclassified_f__Weeksellaceae, and uncultured_f__uncultured_o__Rickettsialess) in spring was found in *R. amurensis* (LDA > 4, *P* < 0.05; Figure 5A).

Thirty-four phyla were identified in *R. dybowskii* in different seasons, and 5 phyla (Actinobacteria, Campylobacterota, Desulfobacterota, Firmicutes, and Verrucomicrobia) showed significant differences (Kruskal-Wallis test, *P* < 0.05). At the phylum level, significant enrichment of two phyla (Desulfobacterota and Firmicutes) in summer, and of two phyla (Actinobacteriota and Verrucomicrobiota) in autumn, and one phylum (Campylobacterota) in spring was found in in in *R. dybowskii* (LDA > 4, *P* < 0.05; Figure 5B). Of all 641 genera in *R. dybowskii*, 62 showed significant differences among different seasons (Kruskal–Wallis test, *P* < 0.05). At the genus level, significant enrichment of six genera (*Anaerotruncus*, *Gordonibacter*, unclassified_f__Erysipelotrichaceae, unclassified_f__Lachnospiraceae, unclassified_f__Ruminococcaceae, and unclassified_o__Oscillospirales) in summer, and of two genera (*Akkermansia* and unclassified_f__Micrococcaceae) in autumn, and three genera (*Deefgea*, *Faecalitalea*, and *Pseudomonas*) in spring was found in in in *R. dybowskii* (LDA > 4, *P* < 0.05; Figure 5B).

### Core microbiota

Among the 2,371 OTUs identified, 116 OTUs were found in all six groups (Supplementary Figure 4A). Ten OTUs were found in all animals (found in >90% of all 52 frogs) with an average relative abundance of 28.72 ± 12.01% of the total relative abundance (Supplementary Figure 4B). The 10 core OTUs came from Bacteroidetes (2), Firmicutes (3), and Proteobacteria (5). The dominant genera were *Pseudomonas*, *Hafnia-Obesumbacterium*, *Citrobacter*, and *Erysipelatoclostridium* (Supplementary Figure 5A). In the as and ds groups, 11 OTUs (present in >90% of all 14 frogs) were found, accounting for 15.66 ± 9.01% and 32.94 ± 25.01% of their relative abundance, respectively (Supplementary Figure 4C). The 11 core OTUs came from Actinobacteriota (3), Bacteroidetes (1), Firmicutes (4), and Proteobacteria (3). *Hafnia-Obesumbacterium*, *Citrobacter*, and *Gordonibacter* dominated (Supplementary Figure 5B). A total of 23 OTUs (found in >90% of all 28 frogs) were found in the ah and dh groups, accounting for 47.49 ± 3.84% and 63.17 ± 3.69% of the total relative abundance in the ah and dh groups, respectively (Supplementary Figure 4D). The 23 core OTUs came from Actinobacteriota (2), Bacteroidetes (5), Firmicutes (7), and Proteobacteria (8). The dominant genera were *Bacteroides*, *Pseudomonas*, and *Deefgea* (Supplementary Figure 5C).

### Predicted functional analysis

**FIGURE 6:**
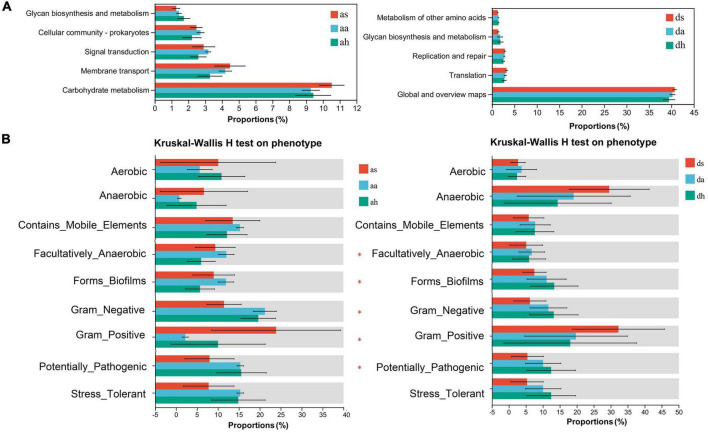
Functional analysis was predicted using PICRUSt2, and bacterial phenotypes were identified using the BugBase method. Panel **(A)** shows that the relative abundance of predicted genes associated with the level-2 KEGG pathway differed significantly in the macro genome. The lists on the right show the level 2 KEGG pathways, and the abundance of each functional route, respectively. **(B)** To determine whether changing seasons have an effect on the function and phenotype of the gut microbial population, bacterial phenotypes were analyzed and predicted using the BugBase algorithm. Significant group differences are denoted by asterisks.

The predominant functions of the gut microbiota in these two *Rana* species were metabolism, genetic information processing, and environmental information processing (Figure 6A). In total, 46 gene families were found in the gut microbiota of *Rana* and were focused on carbohydrate metabolism, Global and overview maps, Glycan biosynthesis metabolism, membrane transport, and replication and repair, translation (Figure 6A). Five functional pathways (Carbohydrate metabolism, Cellular community-prokaryotes, Glycan biosynthesis and metabolism, Membrane transport, and Signal transduction) were found with significantly different abundances among seasons in *R. amurensis* (Figure 6A). Predictions made using PICRUSt2 suggested that functions related to the Global and overview maps, Glycan biosynthesis and metabolism, Metabolism of other amino acids, Replication and repair, and Translation were significantly different between the beginning and end stages of hibernation in *R. dybowskii* (Figure 6A).

To further understand the differential changes in gut microbiota among seasons, we analyzed bacterial phenotypes and used the BugBase algorithm to predict bacterial phenotypes to investigate whether different seasons have an impact on the function and phenotype of the gut microbial population, Anaerobic, Contains_Mobile_Elements, Facultatively_Anaerobic, Forms_Biofilms, Gram_Negative, Gram_Positive, Potentially_Pathogenic, Stress_Tolerant (Figure 6B). There was a similar trend in these two *Rana* species. Among seasons in the *R. amurensis* group, the Facultatively_Anaerobic, Forms_Biofilms, Gram_Negative, Gram_Positive, Potentially_Pathogenic were significantly different. However, there was no difference for *R. dybowskii* (Figure 6B).

## Discussion

### Alpha diversity

Alpha diversity, the most prevalent gut microbiota health indicator, is linked to disease (Zarrinpar et al., 2018). In the present study, alpha diversity fluctuated among seasons, but autumn and spring had no significant differences. Previous investigations of the gut microbiota of hibernating animals have yielded contradictory results in terms of alpha diversity (Weng et al., 2016; Wiebler et al., 2018; Xiao et al., 2019; Zhou et al., 2021; Fan et al., 2022; Greene et al., 2022), which may be due to several factors (such as diet, habitat and temperature). Diet has a significant impact on these communities, with both short-term dietary changes and long-term dietary patterns having an impact (Lee et al., 2020). *Fejervarya limnocharis* in spring, summer, autumn, and prehibernation to study gut microbiome and feeding behavior. Seasonal eating altered gut microbial composition and diversity (Huang and Liao, 2021). Previous studies have compared the differences in intestinal microbiota between hibernating fasted and active feeding animals, rather than comparing both during periods of fasting (Chang et al., 2016). The effect of habitat on gut microbiota is significant (Qing et al., 2019; Tong et al., 2020a). Animals that live in different seasons live in different habitats; for example, *R. dybowskii* lives in terrestrial habitats in summer and hibernates in water (Qing et al., 2019; Tong et al., 2020a). This study was carried out in the same habitat in autumn and spring, but not in summer.

Temperature plays a key role in regulating microbial activity and growth (Sepulveda and Moeller, 2020; Huus and Ley, 2021). Hibernating and normally fed frogs are different in body temperature (Kohl and Yahn, 2016; Weng et al., 2016; Wiebler et al., 2018; Huus and Ley, 2021). The work was carried out under almost identical temperature and environmental conditions. The analyzed periods occurred at the beginning and end of hibernation, unlike prior seasonal research (Weng et al., 2016; Wiebler et al., 2018; Tong et al., 2020b). The body temperatures of frogs in different seasons differ by approximately 10–20°C (Weng et al., 2016). Such temperature variations may have repercussions for the transmission and evolution of intestinal bacteria (Kohl and Yahn, 2016; Huus and Ley, 2021). The gut microbiota of some animals varies considerably with season (Weng et al., 2016; Wiebler et al., 2018; Tang et al., 2019; Tong et al., 2020b; Zhou et al., 2021). However, it is thought that these changes in gut microbiota primarily reflect interpersonal changes in host diet and possibly also temperature, yet the temperature-induced changes in specific gut microbiota found in animal experiments are rarely observed interseasonally in wild hosts (Kohl and Yahn, 2016; Sepulveda and Moeller, 2020).

### Beta diversity

Beta diversity can be a useful indicator for assessing the impact of environmental change (Jackson and Ultsch, 2010). In the present study, the summer, autumn, and spring gut microbiotas of both species differed, as did the autumn (short-term fasting) and winter (long-term fasting) microbiomes. The effect of the seasons on the intestinal microbiota of animals has been studied extensively (Weng et al., 2016; Wiebler et al., 2018; Xiao et al., 2019; Zhou et al., 2021; Fan et al., 2022; Greene et al., 2022). The intestinal microbiota of difference between short-term (weeks, autumn) and long-term (months, spring) fasting has rarely been examined, and there are numerous plausible explanations. Gut microbiotas are greatly affected not only by the feeding status or fasting status but also by the time of fasting (Sonoyama et al., 2009). The effects of fasting time in hibernating frogs on the animal’s body, such as changes in intestinal tissue structure during hibernation, affect the intestinal microbiota (Cerri et al., 2016).

Fasting, which is the temporary cessation of all food intake, can also alter the gut microbiota (Tang et al., 2019). In the present study, the two frog species fed on insects and gradually moved from a feeding state to a fasting state, followed by hibernation. This gradual change from a protein-rich eating state to a fasting state is enabled by their gut microbiota, and this time period corresponds to the gut characteristics during early hibernation (Tang et al., 2019). Fasting has an impact on the gut microbiota and gastrointestinal system, which are both impacted by unabsorbed nutrients and microbial activity (which depends heavily on the host diet for metabolic substrates) (Sommer et al., 2016). Instead of simple carbohydrates, the gut microbiota uses substrates (such as amino acids and glycoproteins) during the feeding phase (e.g., galactose, fucose, and glucose) (Dill-McFarland et al., 2014). In the microbiota, fasting during hibernation causes the loss of taxa that depend on complex plant polysaccharides and the selection of taxa that can degrade and use food derived from the host (Sommer et al., 2016; Carey and Assadi-Porter, 2017). Fasting-induced changes in the gut microbiota may encourage changes brought on by hibernation in the host’s immune system, function of the epithelial barrier, and other aspects that could influence the makeup of the gut microbiota (Carey and Assadi-Porter, 2017).

Amphibian digestive organs undergo extensive structural and functional changes during adaptation to changed physiological and nutritional states during dormancy, which may result in a shift in gut microbiota (Wiebler et al., 2018; Tong et al., 2020b). In this study, the temperature, fasting, and metabolism may have remained the same, but the gastrointestinal function and mass of the frogs changed significantly throughout their 6-month hibernation (Secor and Carey, 2016). Under unpredictable conditions, regulation within organ systems requires a match between energy expenditure and functional demand in a tissue and should be modestly plastic, which is a necessity for organisms to adapt to different environments (Carey and Assadi-Porter, 2017). The gut is the first organ system under the direct influence of dietary changes and is very plastic to variable dietary conditions (Cramp and Franklin, 2005). Fasting deprives the gut of luminal nutrients, causing anatomical atrophy and functional alterations (Tang et al., 2019). During hibernation, the gut’s bulk, villus height, and enterocyte turnover diminish (Cramp and Franklin, 2005; Sonoyama et al., 2009). The intestinal villi, the greatest animal-environment interaction region, store many gut microorganisms and are crucial for immune function (Purchiaroni et al., 2013). Gut microbes are a part of innate immunity in animals and spread massively along the inner gut walls, where they form a layer of the immune barrier against external pathogens (Purchiaroni et al., 2013). Formation of the mammalian immune system depends on special immunoregulatory molecules provided by symbiotic bacteria. For instance, the polysaccharides produced by *Bacteroides fragilis* affect immunomodulation of the host, promote formation of the immune system and maintain the normal roles and TH1/TH2 balance of T cells and lymphoid organs (Dongarrà et al., 2013).

### Differences in gut microbiota compositions

The phyla with abundances above 1% in the late stages of hibernation were Bacteroidetes, Proteobacteria, Firmicutes, and Actinobacteria in both *R. amurensis* and *R. dybowskii* in the autumn and spring. In the spring (the late stages of hibernation), the numbers of bacterial species decreased in both *Rana* species, and the compositions were similar. These four phyla are also ubiquitous gut microbes of birds (Hird et al., 2015), other amphibians (Kohl et al., 2013; Chang et al., 2016), fish (Romero et al., 2014; Liu et al., 2016), and mammals (Sommer et al., 2016). The high abundances of the four phyla may be associated with their important metabolic functions because they have formed stable symbiotic relationships and dominated the gut microbiota compositions in animals during long-term evolution (Shapira, 2016). Microbial composition and species richness favorably affect ecosystem functioning (Bell et al., 2005). In this study, a basic role of the bacterial population during hibernation was revealed by the decline in bacterial species. Because taxa less able to breakdown or utilize host-derived substrates gradually become less numerous until diets appear again during the late stages of hibernation, competition for the scarce resources in the hibernator’s dietary gut may be a significant shaper of the microbiota (Tang et al., 2019).

In the present study, the most dominant phylum was Proteobacteria, and the major representative genus was *Pseudomonas*, whose abundance increased non-significantly after winter. Some species of *Pseudomonas* were isolated from the guts, including *Pseudomonas_sp._MYb218*, *P. azotoformans*, *P. fragi* and four unclassified species. *Pseudomonas* is a diverse genus with various metabolic functions, ecological ubiquity and adaptability to broad environmental niches (Silby et al., 2011). In aquaculture, *Pseudomonas* strains have been used as probiotics to strengthen the pathogenic responses of different hosts (Pérez et al., 2010). However, some species of *Pseudomonas* are pathogenic to fish, shrimp and crustaceans and can infect humans and induce acute diarrhea (Gauthier, 2015). The gut microbiota is delicately balanced, and disruption of this balance leads to dysbiosis and overgrowth of pathobionts, leading to pathologic immune responses and disease (Kamada et al., 2013). *Yersinia*, *Pseudomonas* and *Aeromonas* from Proteobacteria are gut pathogens in humans and animals and may interfere with human health by inducing diarrhea, dysentery and infection (such as necrotic and haemorrhagic disease) (Gauthier, 2015).

### Characteristics of core and shared bacterial communities

The core microbiome provides information on how bacterial taxa may functionally contribute to the host in a particular temporal or dietary situation (Shade and Handelsman, 2012; Risely, 2020). Both frog species had 23 core OTUs in spring (end hibernation), which were higher in number and abundance than the summer core microbiota. These fundamental groupings in the gut of frogs might be indicative of species that are especially resilient to the dramatic nutritional transition between feeding and fasting (Risely, 2020; Perlman et al., 2022). In general, the microbiotas of *R. dybowskii* and *R. amurensis* were very similar at the genera level and higher because the two species inhabited very similar environments and maintained the same minimal body temperatures during hibernation. This phenomenon suggests that a core microbiota may exist among obligate hibernators (Carey and Assadi-Porter, 2017).

The core genus with the highest proportion was *Bacteroides* in in spring (end hibernation). This genus may therefore be critical for hibernation. *Bacteroides* promotes polysaccharide breakdown, efficient food utilization, intestinal mucosal vascularization, immune system development and enteritis prevention, and intestinal microbial balance in animals (Bäckhed et al., 2012; Tang et al., 2019). *Bacteroidetes* is relatively abundant not only during hibernation but also during the fasting stage of Asian seabass, toads and mice (Tang et al., 2019). In this study, the two frog species fasted to the empty state of the early stages of hibernation; thus, the abundance of Bacteroidetes in the two frog species intensified and increased further during the 6-month hibernation period. *Bacteroides* has many benefits for the animal body (Huang and Liao, 2021). In particular, during hibernation, *Bacteroides* may play important roles in maintaining the ecological balance between communities and contributing to the health of the host. It is known that the gut microbiota of amphibians plays a vital role in sustaining their general health and wellbeing. By examining the microbiome of amphibians during hibernation, it is possible to identify the dominant/core microbial communities and assess how they evolve over time. This can assist discover certain bacterial strains that may be especially favorable to amphibian health and could be employed to enhance the growth and survival of these species through conservation initiatives.

### Alterations in the metabolic function of frog intestinal microorganisms

The gut microbial communities of *Rana* were not randomly constructed but instead executed a series of functions that affected metabolism (Zarrinpar et al., 2018). We found that the predominant functions of the gut microbiota involved carbohydrate metabolism, Glycan biosynthesis metabolism and replication and repair, translation. These results are similar to those of a previous study (Weng et al., 2016). These results indicate that the gut microbiota may play a crucial role in supporting the metabolic needs of amphibians during hibernation (Zhou et al., 2021; Regan et al., 2022). During hibernation, animals rely on stored energy stores to support their decreased metabolism (Tong et al., 2020b). The increased presence of carbohydrate and energy metabolism-related genes in the gut microbiota may assist the breakdown and usage of these energy sources (Venema, 2010). In addition, the increased number of genes involved in amino acid metabolism may have a role in facilitating protein synthesis and tissue repair during hibernation (Jackson and Ultsch, 2010). However, the reference database is based on humans and mammals rather than amphibians and thus should be applied with caution to *Rana* (Langille et al., 2013). Moreover, PICRUSt2 has value, it should be complemented with other methods, such as multiple omics (Sung et al., 2016). Future research should focus on elucidating the functional consequences of these alterations in gut microbiota during hibernation as well as their potential impact on host health and well-being. This can help us spot disease outbreaks and other dangers. This data can help conserve amphibians and prevent disease.

## Conclusion

In conclusion, the effects of hibernation on the gut microbiotas of *R. dybowskii* and *R. amurensis* were investigated via high-throughput Illumina sequencing. In comparison to autumn and winter, the alpha diversity of the gut microbiota was much higher in the summer. Yet, neither of the frog species showed any appreciable variations in diversity between autumn and spring. Both species’ gut microbiotas had different gut microbiome compositions in the summer, autumn, and spring. Between autumn and winter, the microbiome underwent considerable changes. The study of the gut microbiome of hibernating amphibians can provide vital information on their health, well-being, and the risks they experience in the wild. This information can be used to support focused conservation initiatives that encourage amphibian population growth and survival.

## Data availability statement

The datasets presented in this study can be found in online repositories. The names of the repository/repositories and accession number(s) can be found in the article/Supplementary material.

## Ethics statement

The animal study was reviewed and approved by the IACUC of Northeast Agricultural University (IACUC#2015-035).

## Author contributions

QT, Z-FH, and M-DX: data collection, data analysis and interpretation, and drafting the manuscript. QT and L-YC: conception or design of the work, sample collection, and final approval of the submitted version. QT, W-JD, PG, X-YH, and Z-FH: writing and critical revision of the manuscript. All authors contributed to the article and approved the submitted version.

## Footnotes

1
https://bugbase.cs.umn.edu/index.html
