# Supplementary material for: Characteristics and a comparison of the gut microbiota in two frog species at the beginning and end of hibernation
Source: Front Microbiol. 2023 May 3;14:1057398. doi: 10.3389/fmicb.2023.1057398 (PMC10191234; doi:10.3389/fmicb.2023.1057398)
Supplement: Supplementary file 1 [file Data_Sheet_1.doc]

**Additional files**

**Characteristics and a comparison of the gut microbiota in two frog species during** **beginning and end hibernation**

**Qing Tong^1,2,3^*, Wen-jing Dong^1^, Ming-da Xu^1^, Zong-fu Hu^2^, Peng Guo^1^,** **Xiao-yun Han^1^, Li-yong Cui^3^***

1 School of Biology and Agriculture, Jiamusi University, Jiamusi, 154007, China

2 College of Veterinary Medicine, Northeast Agricultural University, Harbin, 150030, China

3 Hejiang Forestry Research Institute of Heilongjiang Province, Jiamusi 154002, China

*** Correspondence:**

Qing Tong, lxixl@126.com

Li-yong Cui, [cuiliy@163.com](mailto:cuiliy@163.com)


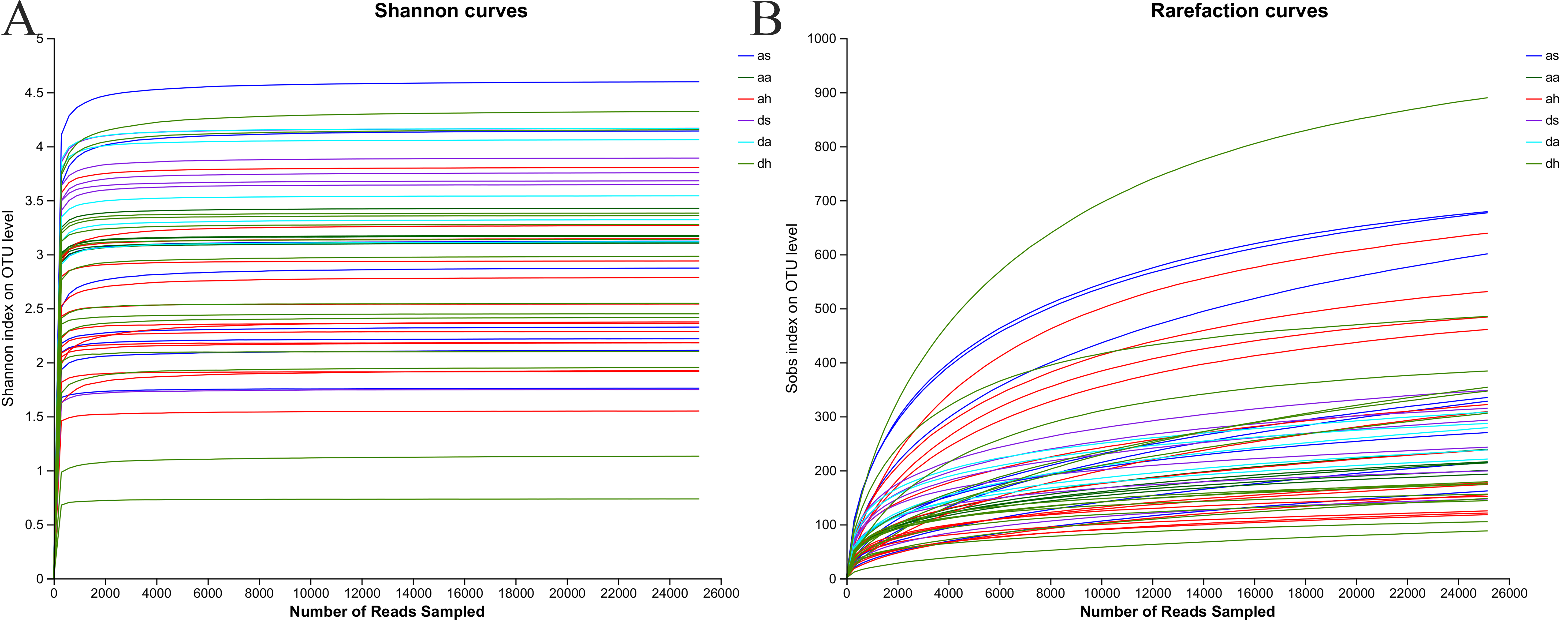


**Figure S1** Rarefaction curves (**A**) and Shannon curves (**B**) of all samples.

The rarefaction curves are plots of the number of OTUs as a function of the number of sequences. The Shannon curves reflect the micro-diversity of the samples.


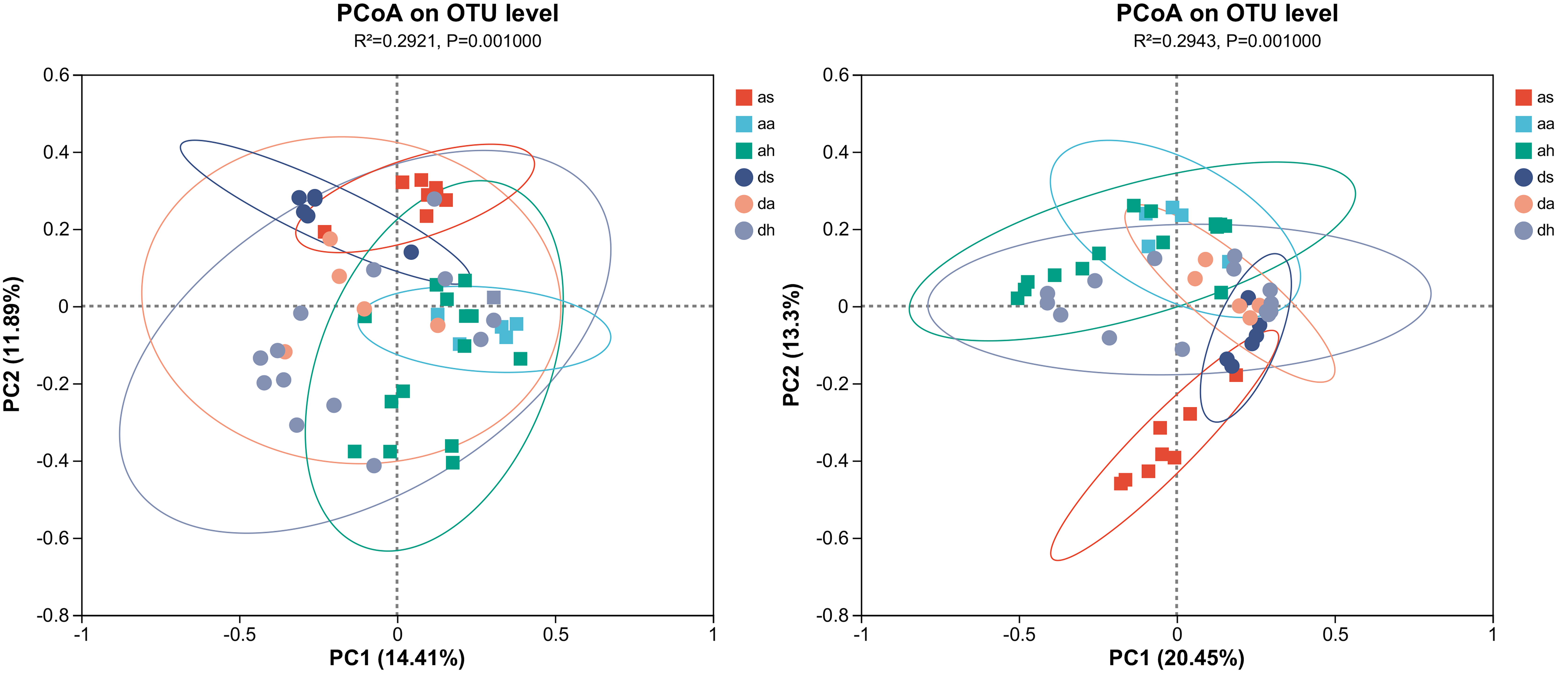


**Figure S2** Gut microbiota differences and similarities.

Principle coordinate analysis (PCoA) indicates separation by seasons based on Bray-Curtis (A) and unweighted UniFrac (B) distances. All OTUs were subjected to PCoA. Each dot represents the gut microbial community of one brown frog.


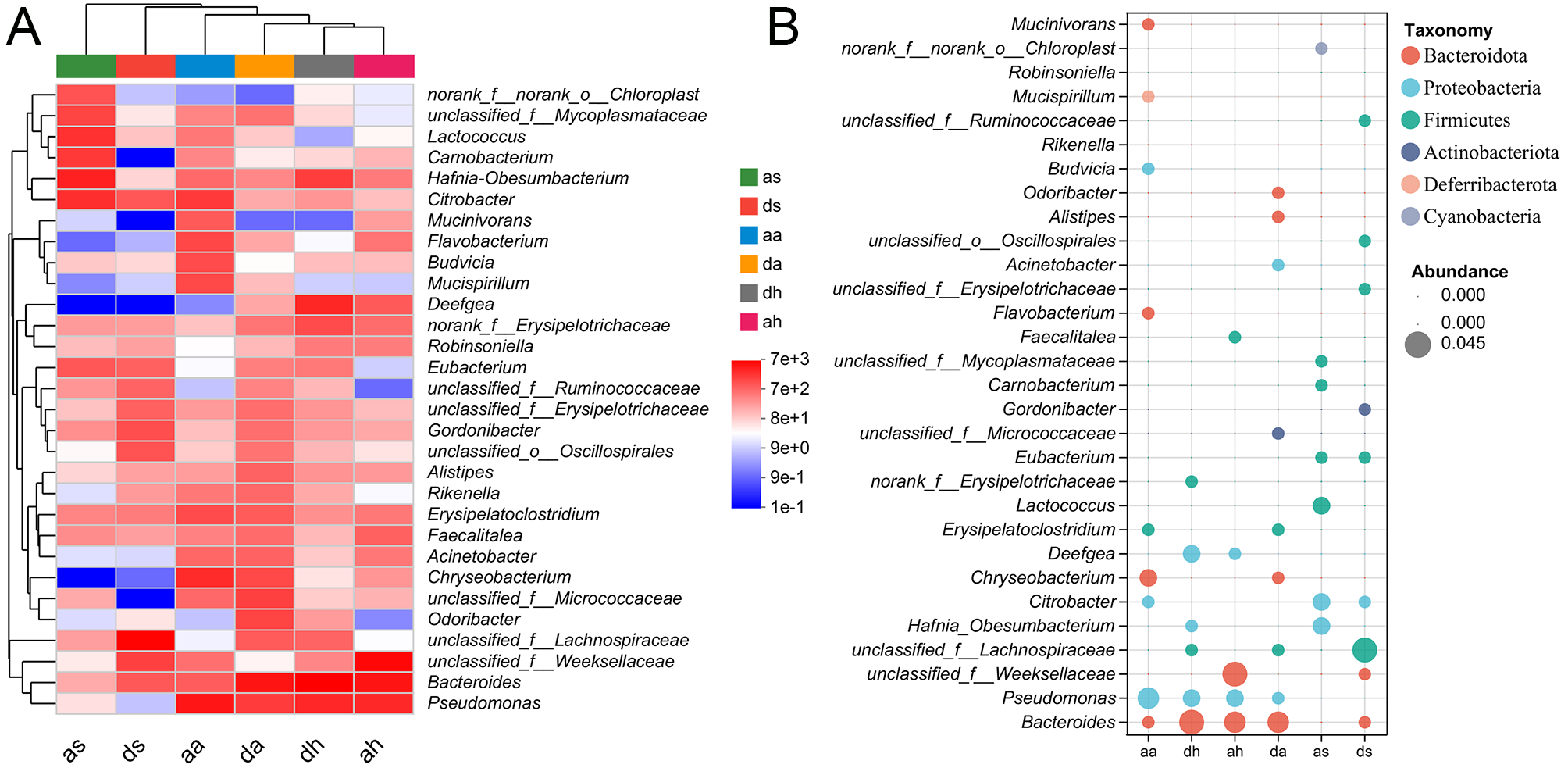


**Figure S3** Heatmap showing the difference between summer, autumn, and spring at the genus level between the two frog species.

Cluster analysis was performed using Bray-Curtis distances and the average-linkage method. Each bar or column corresponds to a specimen. The data are calculated in terms of relative abundance, and the colours represent lg values (A). The values are shown on a scale from green to red, with green representing lower values and red representing greater values (B).


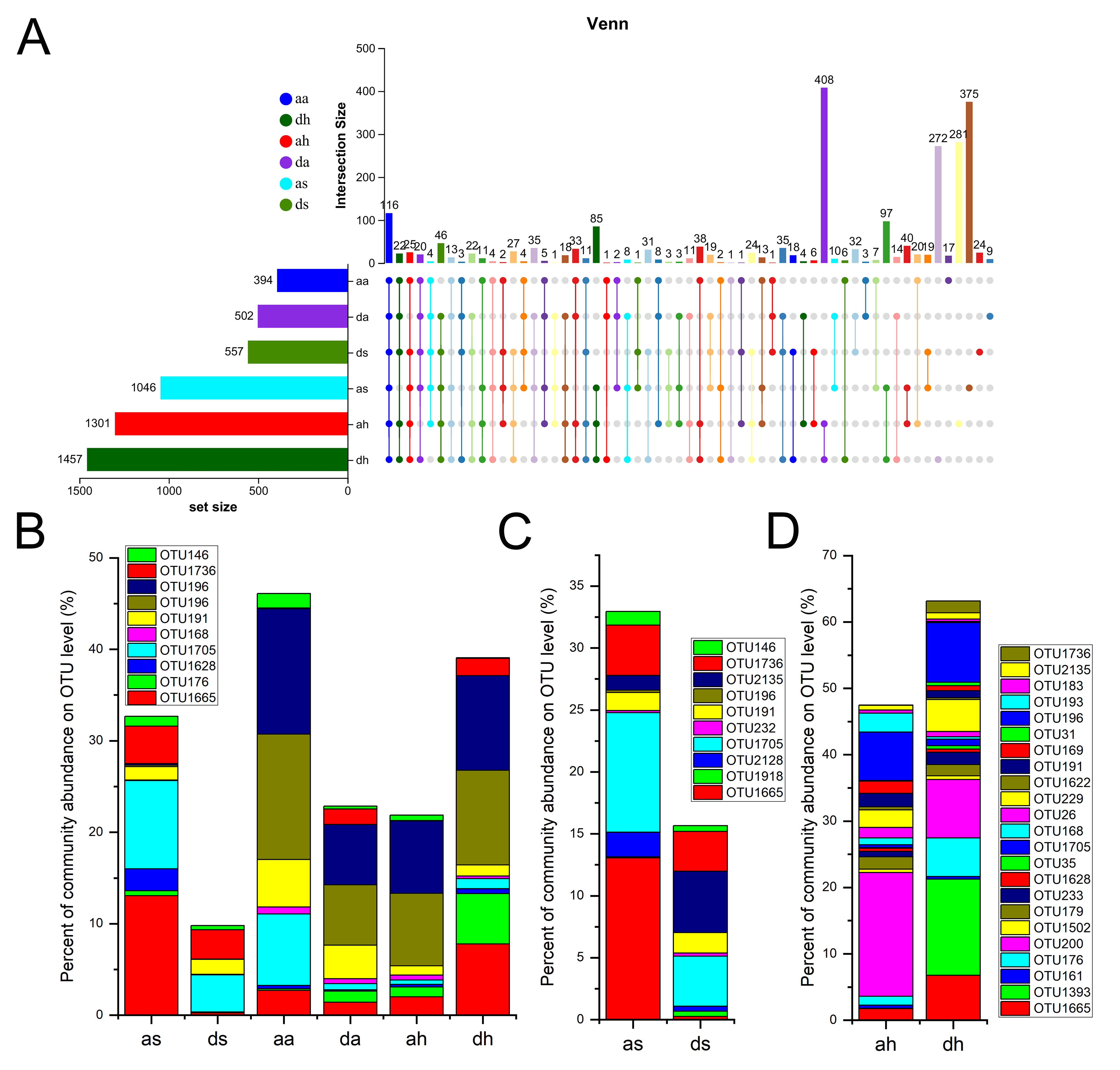


**Figure S4** Core, shared and unique OTUs.

(A) Common and unique OTUs of the gut microbiota in different season groups. Venn diagrams depict the number of OTUs that the summer, autumn, and spring at the OTU level between the two frog species share and have exclusively. Percentage of core OTUs for all frog samples (all groups, B) at the genus level, percentage of core OTUs for both summer frog samples (as and ds groups, C) at the genus level, and percentage of core OTUs for both spring frog samples (ah and dh groups, D) at the genus level. The above ratio is the percentage of core OTUs to all OTUs.


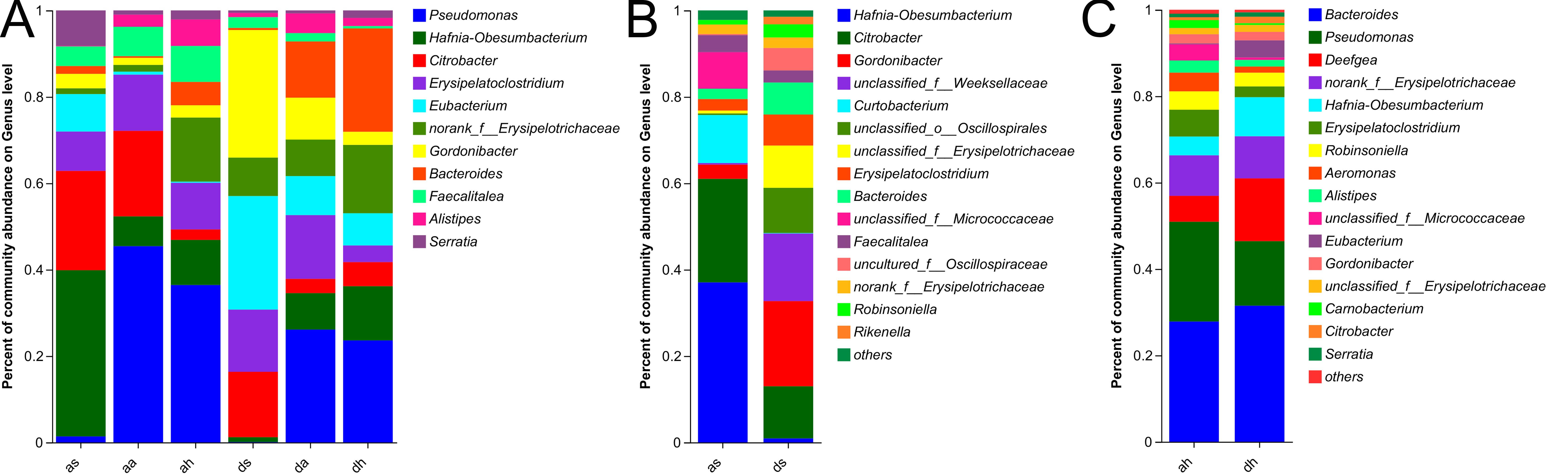


**Figure S5** Percentage of core OTUs for all groups, as and ds groups, and ah and dh groups at the genus level.

Percentage of core OTUs for all samples at the genus level (A), percentage of core OTUs for both summer frog samples (as and ds groups, B) at the genus level, and percentage of core OTUs for both spring frog samples (ah and dh groups, C) at the genus level. The above ratios take the core OTUs as 100%, not all OTUs as 100%.
